# Electrochemical Formation of Pb Microwires with Tunable Morphology on Liquid Metal Electrodes

**DOI:** 10.1021/acsomega.4c09165

**Published:** 2024-10-31

**Authors:** Panjaphong Lertsathitphong, Sarunputt Limpijumnong, Mithran Somasundrum, Anthony P. O’Mullane, Benchaporn Lertanantawong

**Affiliations:** † Biosensors Laboratory, Department of Biomedical Engineering, Faculty of Engineering, Mahidol University, Nakhon Pathom 73170, Thailand; ‡ Biosciences and System Biology Team, Biochemical Engineering and System Biology Research Group, National Center for Genetic Engineering and Biotechnology, National Science and Technology Development Agency at KMUTT, Bangkok 10150, Thailand; § School of Chemistry, Physics and Mechanical Engineering, Queensland University of Technology (QUT), Brisbane, Queensland 4001, Australia

## Abstract

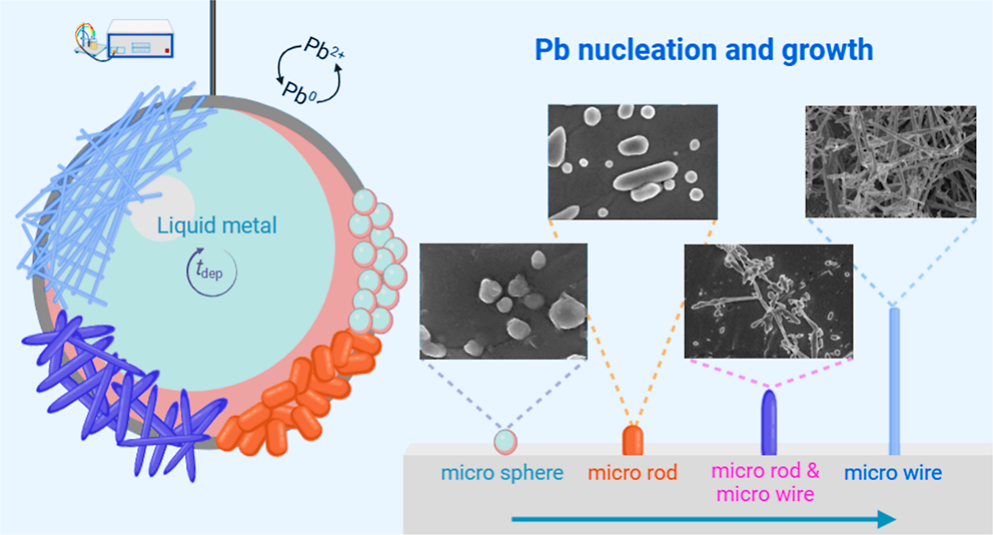

Liquid metal electrodes based on Ga are an emerging area of interest given their fluid properties which can have significant impact on electrochemical processes. Here we study metal electrodeposition, namely lead electrodeposition on the liquid metal electrodes, gallium (Ga) and galinstan (GaInSn), which was performed in two different Pb^2+^ electrolytes (PbCl_2_ and Pb(NO_3_)_2_) to investigate any differences in the nature of the electrodeposit. Cyclic voltammetry and chronoamperometry were used to study the characteristics, kinetics, and nucleation and growth mechanisms of the electrodeposition process. Analysis of this electrochemical data, such as current density-time transients and diffusion coefficients under different potentials, revealed distinct behaviors for Pb deposition at each liquid metal and electrolyte, influencing the final morphology of the lead deposit. It was also found that the electrolyte concentration and deposition time were found to impact the morphology of the electrodeposited Pb. Scanning electron microscopy and energy dispersive X-ray spectroscopy revealed various types of Pb microstructures, including wire, branch-like, and flake-like formations, highlighting the differences in lead structural development when deposited on liquid gallium and Galinstan electrodes.

## Introduction

Liquid metals, such as gallium (Ga) and its alloys have low melting points which are often close to room temperature,^1,2^ making them interesting electrode materials due to their liquid state. From an electrochemical perspective, the traditional liquid metal mercury (Hg) electrode has become limited in its use due its toxicity.^2^ Thus, gallium (Ga), with a melting point of 29.8 °C, has recently emerged as a promising alternative due to its low toxicity and favorable biocompatibility.^3^ The exceptional properties of gallium, such as superb fluidity, low viscosity, and high electrical conductivity,^1,4^ have led to its application in various areas of electronics. These include optoelectronic devices,^5,6^ flexible electronics,^7,8^ and electrodes for use in biomedical applications.^9−12^ In terms of electrochemical applications, Ga and its alloys in the liquid state have been used as electrocatalytically active electrodes^13,14^ as well as used for heavy metal ion detection^15−17^ in an analogous manner to the traditional Hg electrode.

The ability of gallium (Ga) to form alloys with a wide range of metals has also received significant attention.^18^ This versatility has expanded research beyond electronic applications, including CO_2_ and methane conversion to carbon at room temperature,^14,19−23^ fuel cell-related reactions,^24^ polymer synthesis,^25^ photocatalysis,^26,27^ pollutant degradation,^28^ energy storage,^29,30^ ammonia electrosynthesis,^13^ and antimicrobial applications.^31,32^ A key factor in these applications is the composition of the alloy, which determines specific activity, as well as the concentration of the second metal in the liquid Ga matrix. A notable example is the inclusion of a highly dilute source of Pt atoms within liquid Ga. In this liquid state, Pt exhibits exceptional atom utilization efficiency for ethanol electro-oxidation.^24^ Another example is the inclusion of Ce nanoparticles in liquid Ga, creating a cyclic CeO_2_/Ce process that reduces dissolved CO_2_ into solid carbon.^12^ Here, the concentration of Ce determines the activity. Therefore, controlling the solubility of the second metal becomes critical and needs to be adjusted to the specific application.

Concurrently, the exploration of metallic nanowires presents another dimension to materials research. Metallic nanowires, characterized by their elongated structure and high aspect ratio, offer unique electrical characteristics, good mechanical flexibility and high thermal conductivity.^33^ Therefore, diverse applications have been found, including supercapacitor electrodes,^34^ optoelectronic devices,^35^ and flexible and transparent energy devices.^36^ In relation to this study, lead nanowires have been reported to possess properties suitable for applications in nanoelectronics and sensing devices.^37^ Intriguingly, Ga–Pb nanocomposites, formed by combining gallium and lead, exhibit distinct properties from both constituents and phase diagrams have been utilized to better understand the intricacies of the alloy.^38^ Evaporation–condensation techniques in ultrahigh vacuum conditions have been performed to explore the relationship between the nanocomposite’s wet–dry condition and temperature. These investigations provide valuable insights into the structural and functional properties of Ga–Pb nanocomposites.^39^ Recent research into Ga–Pb electrodeposition under alkaline conditions reported the formation of Pb quasi-epitaxial crystals on liquid metals surface. This deposition revealed discrete triangles, irregular coalesced structures of Pb, and a cubic lattice structure with the [111] plane perpendicular to the liquid metal surface.^40^ The formation of ordered metals on the surface of a liquid metal without any order opens up new areas of interest for metal electrodeposition.

This work focuses on the nucleation and growth mechanisms of lead electrodeposition on liquid metal electrodes, specifically targeting gallium and the Galinstan (GaInSn) alloy. This investigation assesses the impact of electrolyte composition, specifically aqueous solutions of PbCl_2_ and Pb(NO_3_)_2_ at different concentrations, on the electrodeposition of Pb on pure liquid metal (gallium) and alloy liquid metal (Galinstan) electrodes. A key aspect of the study is to understand the distinct growth mechanisms of lead nanostructures on each type of liquid metal, ultimately leading to the formation of lead nanowires.

## Experimental Section

### Materials

Liquid gallium (Ga) (≥99.99%) and liquid Galinstan (GaInSn) (≥99.99%) were purchased from rich-metals, China. Lead(II) chloride (Aldrich), lead(II) nitrate (Sigma-Aldrich). A 0.2 M sodium acetate buffer solution (pH 4.5) was prepared using sodium acetate anhydrous (Sigma) and acetic acid (AR grade, RCI Labscan). This buffer was used as the supporting electrolyte for all electrochemical measurements. The buffer were prepared with deionized water (resistivity of 18.2 MΩ cm at 25 °C, Milli-Q IQ-7000, Merck).

### Electrochemical Measurements

A three-electrode system was used to deposit lead on a liquid metal hanging drop working electrode (3 mm diameter of gallium or Galinstan drops) with a silver/silver chloride [Ag/AgCl (3 M KCl)] reference electrode and a platinum coil counter electrode. All electrochemical experiments were carried out with an Autolab PGSTAT302N potentiostat using NOVA version 1.11 software (Metrohm, Netherlands). The lead nanostructures on the liquid metal surface were electrodeposited by chronoamperometry (various potentials and times) in the 0.2 M sodium acetate buffer solution (pH 4.5) which contained either various concentrations of PbCl_2_ or Pb(NO_3_)_2_. The transient data were recorded over a period of 30 s for each applied potential. Cyclic voltammetry was used to investigate the electrochemical behavior of Pb^2+^ on the liquid metal surface over a potential start at 0 sweep to −1.0 V at a scan rate of 0.05 V s^–1^. The charge was calculated by integrating the area under the cyclic voltammogram curves, with the reduction charge taken as the area below the zero-current line and the oxidation charge as the area above it. The liquid metal was changed to a fresh liquid metal for every measurement to avoid side reactions. Prior to electrochemical experiments all solutions were deaerated with purified nitrogen to remove oxygen for at least 5 min. The choice of pH 4.5 for the sodium acetate buffer in our electrodeposition solution was based on several key factors. At this pH, lead ions (Pb^2+^) maintain sufficient solubility, ensuring a high concentration of electroactive species for efficient deposition. Importantly, this pH also minimizes the rate of hydrogen evolution, a competing reaction that can interfere with lead deposition. Our choice aligns with established practices, as sodium acetate buffer at pH 4–5 is commonly used in stripping voltammetry methods for lead analysis on various electrodes.^41,42^ Previous studies have investigated lead film deposition using electrochemistry in the pH range of 3.5–6.1,^43^ supporting our selection.

### Characterization

The morphology and elemental analysis of lead on the liquid metal surfaces were determined by a field emission scanning electron microscope, using a JSM-7610 FPlus (JEOL) with energy-dispersive X-ray spectroscopy (EDS) detector (Ultim Max, Oxford Instruments) at an operating voltage of 10 kV for observing the morphology and 20 kV for the EDS mode under high-vacuum conditions. The scanning electron microscopy (SEM) samples were coated with platinum using an Auto Fine Coater model JEC-3000FC (JEOL). Multiple SEM images were taken across different areas of each sample and from multiple deposition experiments to ensure the observed structures were representative. While small variations in the appearance of the deposits were noted, the overall morphological trends described were consistent across samples.

## Results and Discussion

### Study of Lead Electrodeposition Using Liquid Metal Electrodes

**Figure 1:**
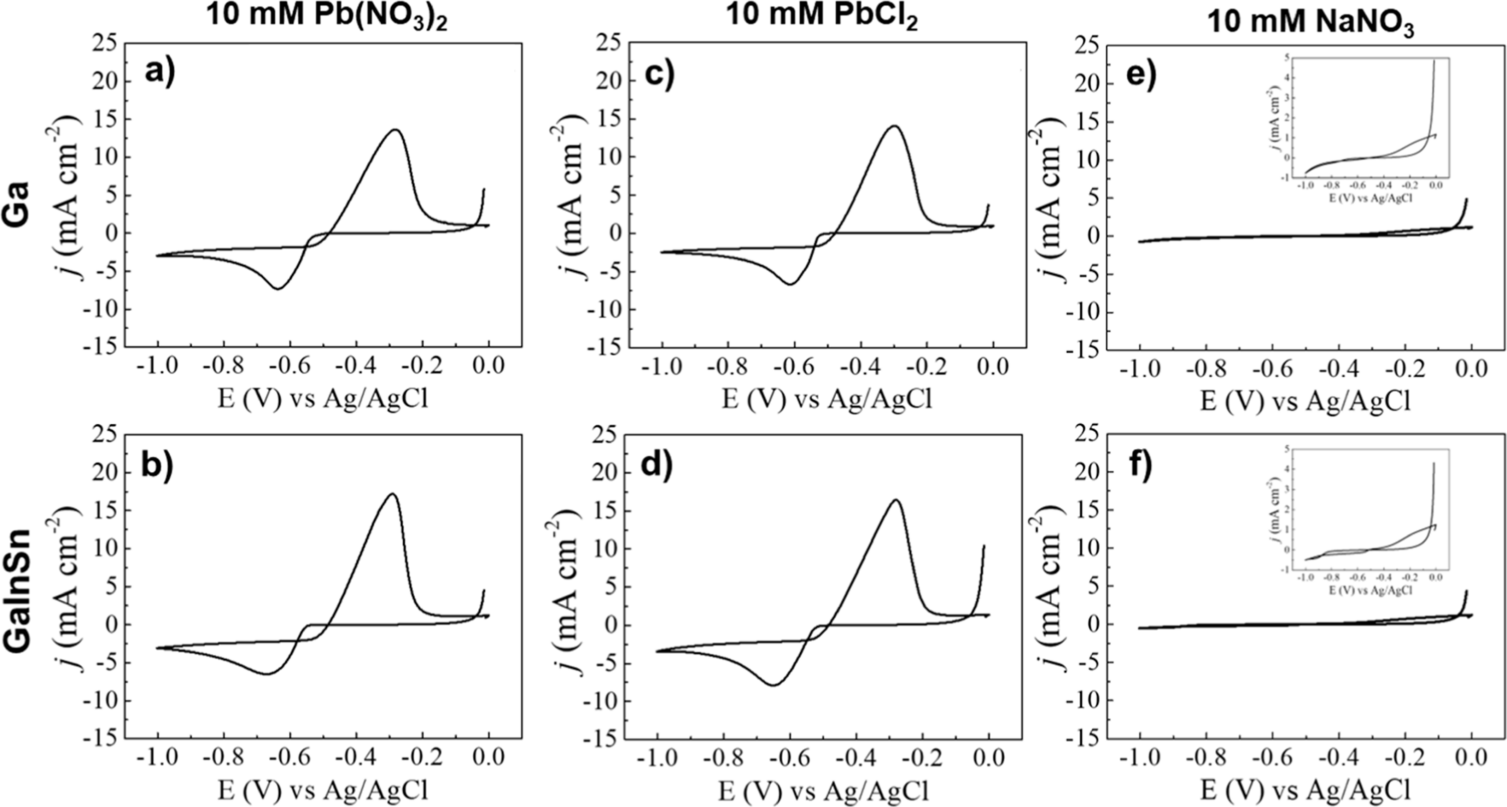
Cyclic voltammograms from a 0.2 M sodium acetate buffer pH 4.5 containing (a,b) 10 mM Pb(NO_3_)_2_ on (a) gallium (Ga) and (b) Galinstan (GaInSn) electrodes. The voltametric behavior of (c,d) 10 mM PbCl_2_ on (c) Ga and (d) GaInSn electrodes and from (e,f) 10 mM NaNO_3_ on (e) Ga and (f) GaInSn electrodes using the zoom in insert.

The electrochemical behavior of a liquid metal working electrode, in the presence of either 10 mM PbCl_2_ or Pb(NO_3_)_2_ was initially studied using cyclic voltammetry, the powerful technique for characterization and restructuring electrode through electron transfer.^44,45^ Cyclic voltammograms were recorded at a scan rate of 50 mV s^–1^ in a 0.2 M acetate buffer electrolyte to compare the electrochemical responses of two liquid metal hanging drop electrodes—Gallium and Galinstan—in two different Pb^2+^ mediums (PbCl_2_ or Pb(NO_3_)_2_) (Figure 1) as well as a control experiment in 10 mM NaNO_3_ only with the background electrolyte. For the control experiment there is very little redox activity in the chosen potential window for both electrodes. A small broad oxidation process at ca. −0.20 V is observed for both electrodes (Figure 1e,f insets) due to Ga-oxidation.

Figure 1 shows quite similar cyclic voltammetric behavior of the two liquid metals in both Pb^2+^ media. There is a cathodic peak for Pb^2+^ reduction to Pb^0^ on the negative sweep followed by a crossover in the current response before the oxidation of Pb^0^ to Pb^2+^ on the positive sweep. In 10 mM Pb(NO_3_)_2_, the redox peak for Pb^2+^ reduction on the gallium electrode (Figure 1a) is observed at −0.635 V with crossover points at −0.483 and −0.552 V followed by the oxidation process at −0.287 V. For the Galinstan electrode (Figure 1b), the reduction and oxidation peak potentials are −0.665 and −0.292 V, respectively with crossover points at −0.485 and −0.578 V. In the PbCl_2_ electrolyte very similar behavior is seen at the reduction/oxidation peak potentials are very similar which can be observed from the data presented in Table 1. As seen in all cyclic voltammograms for lead deposition the current crossover indicates a nucleation loop^46,47^ which is indicative of Pb nucleation and growth kinetics^48^ occurring on the liquid metal electrodes. The reduction process in each case passes more charge than the oxidation process (Table 1). This indicates that not all of the Pb^0^ is stripped from the liquid metal electrodes and that some residual alloy may be present on the liquid metal.

**Table 1:** Anodic and Cathodic Peak Potentials and Charge Associated with Pb^2+^ Reduction and Pb^0^ Oxidation for Lead Electrodeposition on the Liquid Metal Electrodes Ga and GaInSn from a 0.2 M Sodium Acetate Buffer (pH 4.5) Containing Either 10 mM PbCl_2_ or Pb(NO_3_)_2_

reaction	peak potentials (V)	charge (mC)
Ga-10 mM PbNO_3_		
oxidation	–0.282	6.53
reduction	–0.640	–7.51
Ga-10 mM PbCl_2_		
oxidation	–0.292	7.30
reduction	–0.670	–7.91
GaInSn-10 mM PbNO_3_		
oxidation	–0.302	6.28
reduction	–0.614	–6.95
GaInSn-10 mM PbCl_2_		
oxidation	–0.282	7.71
reduction	–0.655	–8.58

### Morphological Observations from SEM Imaging

**Figure 2:**
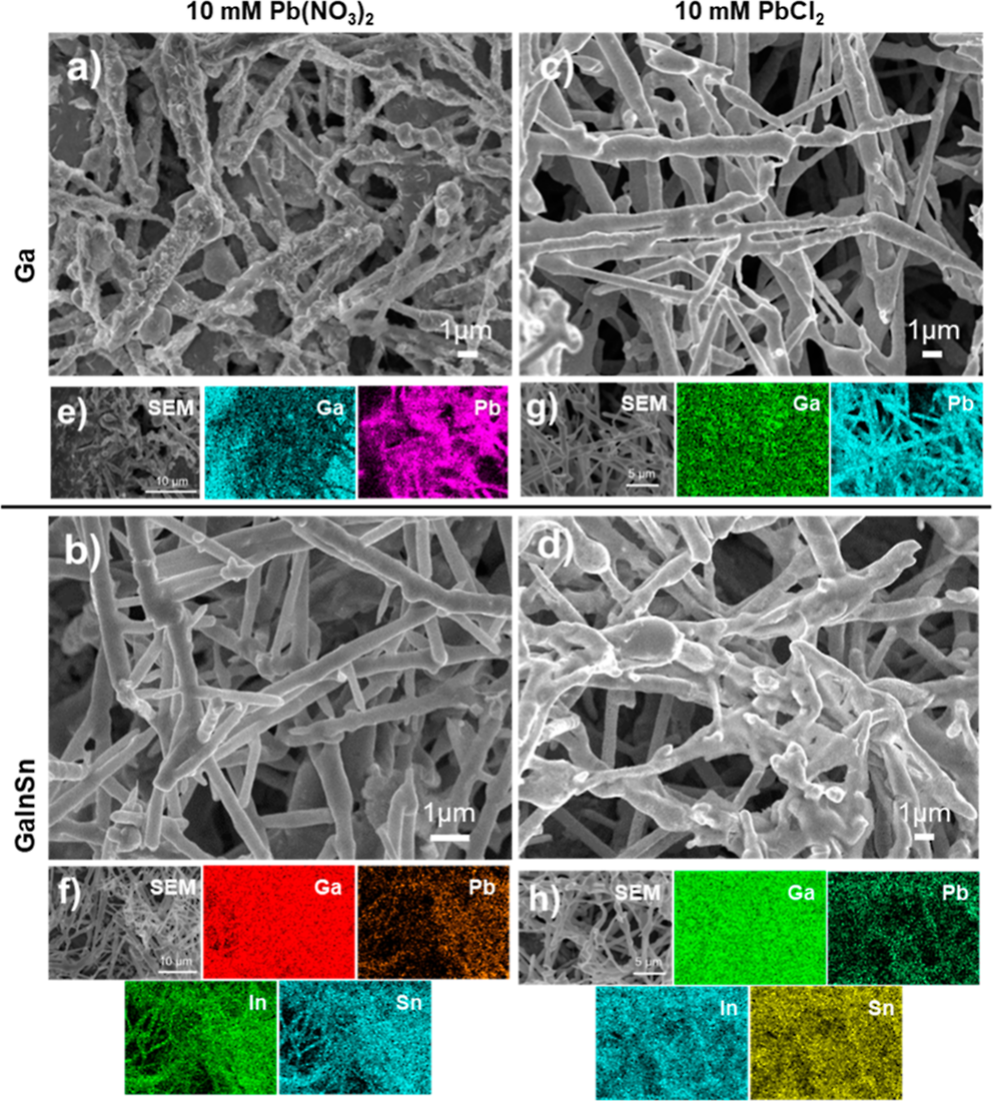
SEM (a–d) and EDS element mapping (e–h) images of lead electrodeposition in a 0.2 M sodium acetate buffer (pH 4.5) containing 10 mM Pb(NO_3_)_2_, using (a,e) Ga and (b,f) GaInSn electrodes, and in PbCl_2_ with (c,g) Ga and (d,h) GaInSn.

To study the morphology of lead electrodeposition a hanging drop liquid metal electrode was employed using an electrodeposition potential of −1.0 V for 300 s. SEM and EDS imaging (Figure 2) for lead electrodeposition from Pb(NO_3_)_2_ and PbCl_2_ onto liquid Ga and Galinstan hanging drop electrodes are mostly consistent in all cases and demonstrates elongated wire structures that are branched and overlap with each other. A control chronoamperometric experiment was performed using 10 mM NaNO_3_ in 0.2 M acetate buffer on a liquid Ga metal electrode. The SEM and EDS mapping images (Figure S1) show a gallium surface without the presence of any particles.

However, the morphology observed for lead deposition on gallium is slightly different in that the branch-like deposits are covered by flake structures that are spread across the surface. This contrasts with the much smoother branches observed on Galinstan. In addition, there is a very slight effect from the anion present in the electrolyte, as seen in the SEM images where the structures formed on gallium that contain Cl^–^ ions (Figure 2c) are wires stacked on top of each other whereas on Galinstan (Figure 2d), the branches coagulate into dense nodes in some areas. To investigate the composition of these wires on the liquid metal surfaces, EDS analysis was conducted (Figure 2e–h). EDS mapping of the microwires electrodeposited on the gallium liquid metal electrode in each lead electrolyte reveals a uniform elemental distribution in the branch-like structures, primarily consisting of Pb when using Pb(NO_3_)_2_ (Figure 2e) and PbCl_2_ (Figure 2g). In the case of lead microwire deposition on the Galinstan electrode, the EDS mapping of the microwires electrodeposited from Pb(NO_3_)_2_ shows an interesting phenomenon in that the microwires show an accumulation of In and Sn elements as well as Pb suggesting trimetallic microwires may have been formed. For the case of PbCl_2_, there is also a uniform dispersion of Ga, In, Sn and Pb across the sample. This indicates that different liquid metals produce distinct characteristics in conjunction with the type of lead electrolyte that is used, even when the morphology is similar.

### Impact of Electrolyte Concentration and Time-Dependent Growth Mechanisms on Lead Microwire Morphology

Different electrodeposition times and metal ion concentration can directly affect film thickness and properties of the metal.^49,50^ Longer deposition times and higher concentrations generally result in more extensive and branched structures and this phenomenon is explored at liquid metal electrodes. A potential of −1.0 V in the diffusion limited region was chosen and applied to the liquid metal electrodes for 300 s at different lead salt concentrations ranging from 2 to 20 mM, facilitating lead deposition on their surfaces. The cyclic voltammetric behavior for different concentrations of PbCl_2_ at Ga and GaInSn electrodes is presented in Figure S2 and described in the Supporting Information. SEM images of lead electrodeposition at both gallium and Galinstan electrodes from 2 mM PbCl_2_ (Figure S3e,f,o,p) show a transformation from a smooth to a rough surface, attributed to the formation of layers and sheet-like structures that reshape the liquid metal surface. In higher concentrations of PbCl_2_ (10 and 20 mM), SEM images reveal two distinct morphologies (Figure S3c,d,m,n): the rough surface similar to lower concentration deposition and a branch-like structure. Notably, the morphology of lead–gallium and lead–Galinstan electrodeposited from 20 mM PbCl_2_ exhibits different structures; gallium presents long branch-like structures and rough surfaces (Figure S3a,b), whereas Galinstan shows short branch-like and sheet structures (Figure S3k,f).

The restructuring effects on gallium from lead deposition in varying concentrations of Pb(NO_3_)_2_ were also observed through SEM images. For 2 mM Pb(NO_3_)_2_, the images (Figure S3i,j) depict spherical liquid metal particles with flake-like structures on the surface, alongside fractal-like features. These structures, while similar in morphology, display different sizes and branching patterns. Increasing the Pb(NO_3_)_2_ concentration to 10 mM reveals a mix of larger branch-like particles and smaller flake-like structures. The surfaces of these branch-like particles are covered with sheet particles and adorned with small flake-like structures, creating a spike-like appearance (Figure S3g,h). For lead electrodeposition in Galinstan from Pb(NO_3_)_2_, SEM images at 2 mM concentration (Figure S3s,t) show a rough surface with small spherical particles. However, at a higher concentration of 10 mM (Figure S3q,r), the structure transitions to a branch-like formation.

**Figure 3:**
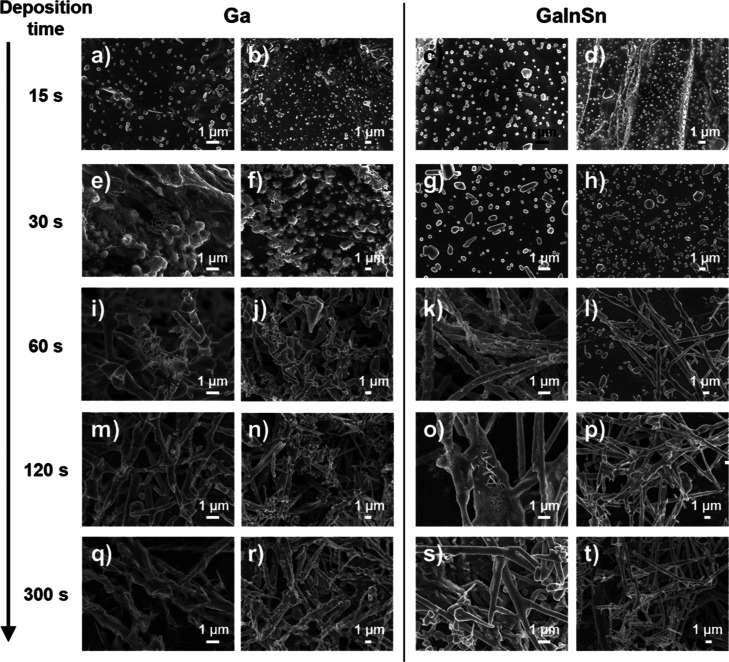
SEM images of lead electrodeposition from a 0.2 M Sodium acetate buffer (pH 4.5) containing 10 mM Pb(NO_3_)_2_ on Ga liquid metal electrodes over varying deposition times: (a,b) 15, (e,f) 30, (i,j) 60, (m,n) 120, and (q,r) 300 s; and on GaInSn liquid metal electrodes over (c,d) 15, (g,h) 30, (k,l) 60, (o,p) 120, and (s,t) 300 s.

The effect of electrodeposition time was then investigated to monitor the microwire growth process. SEM images reveal the initial structures that are formed during the first 15 s of lead deposition on both gallium (Figure 3a,b) and Galinstan electrodes (Figure 3c,d) where spherical and oval shaped structures are observed on the surfaces of both liquid metals with the emergence of some short, elongated structures. Upon extending the deposition time to 30 s, distinct morphological differences become evident between the two liquid metals. On gallium (Figure 3e,f), the lead forms spike-like and net-like structures that emerge from the spherical seeds. In contrast, on Galinstan (Figure 3g,h), the lead exhibits the formation of more isolated elongated structures that are longer than after 15 s of deposition but are still disconnected from each other. Upon further extending the deposition time to 60 s (Figure 3i–l) the formation of longer wire structures as well as small clusters of lead wires is evident. However, this bunching of wires is much more pronounced on the liquid Ga electrode. When the deposition times reach 120 and 300 s (Figure 3m–t), similar morphologies are observed, characterized by microwires with flake-like or spike-like structures on the gallium surface, and smoother microwires on the Galinstan surface. The growth mechanisms on each liquid metal differ whereby the lead microwires on gallium grow by forming spikes on the surface, while on Galinstan, they elongate from the initial spherical seed particles that are formed. This growth pattern resembles the mechanism observed in the growth of single-crystalline lead nanowires.^37^ The growth of lead microwires progresses through four steps; (i) the initial reduction of lead ions to Pb^0^ (ii) seed formation which leads to the formation of small lead nanoparticles (iii) microcrystals growth; some nanoparticles continue to grow into larger microcrystals (iv) microwire growth; lead wires nucleate and grow from the corners of the microcrystals by spherical-particles at the top of the wire, consuming smaller nanoparticles through Ostwald ripening. In addition, the growth process of lead wires resembles a solution–liquid–solid (SLS) process.^37^ To illustrate the uniformity of the deposits that are achieved, additional SEM images are provided in Figure S4 while optical images of the GaInSn hanging drop electrode are presented after different electrodeposition times from a solution containing 10 mM Pb(NO_3_)_2_ (Figure S5). It can be seen that the initial mirror like appearance of the liquid metal electrode turns uniformly gray, followed by a uniform dark deposit after 300 s where larger protrusions from the surface can be seen.

### Analyzing Kinetic Parameters of Lead Electrodeposition onto Gallium and Galinstan Electrodes

**Figure 4:**
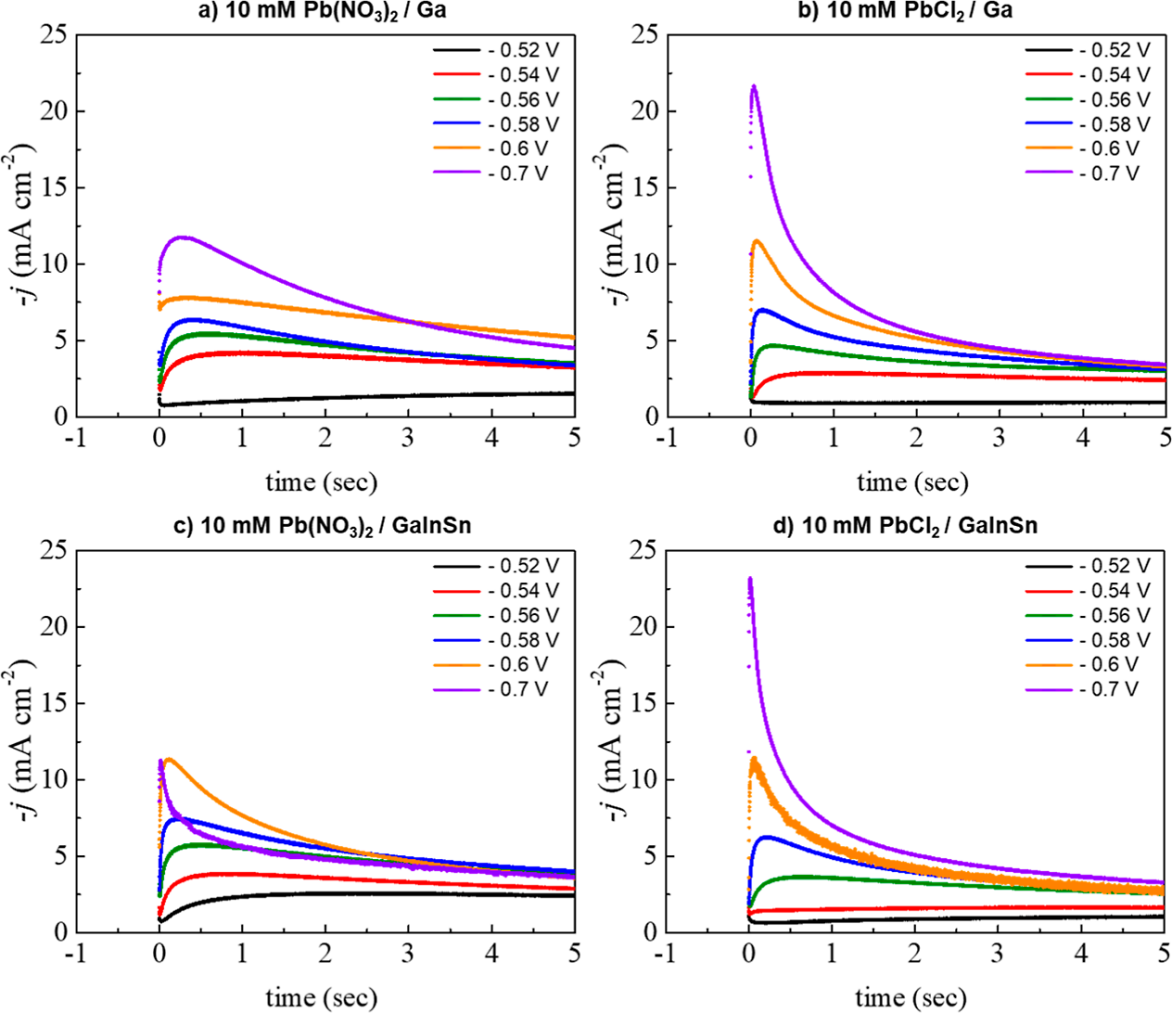
Transients current density data for lead electrodeposition on Ga and GaInSn liquid metal electrodes from a 0.2 M sodium acetate buffer (pH 4.5) containing either 10 mM PbCl_2_ or Pb(NO_3_)_2_.

The electrochemical mechanism for lead electrodeposition was analyzed using chronoamperometry on gallium and Galinstan liquid metal electrodes from 0.2 M sodium acetate buffer (pH 4.5) containing either 10 mM PbCl_2_ or Pb(NO_3_)_2_ using potential steps over a potential range of −0.52 to −0.70 V as shown in Figure 4. These types of transient curves typically exhibit three phases,^46^ namely, an initial small descending current decay caused by double layer charging and the formation of the first nuclei on the substrate’s surface, an ascending part where the current density rises to a maximum (*j*_max_) at a given time (*t*_max_) due to nucleation and growth, and a longer descending part caused by higher nucleation density interference in the diffusion process.

The electrochemical behavior of lead deposition on both liquid metal surfaces with different lead electrolytes follows this pattern; an increase in the current passed accompanied by a decrease in the time required to reach the maximum current (*t*_max_) with increasingly negative deposition potential. This is typical of diffusion-controlled behavior for metal electrodeposition, which exhibits a shorter rising portion upon increasing the overpotential.^51^ It is worth noting that the current density for lead deposition differs between PbCl_2_ and Pb(NO_3_)_2_ electrolytes. At −0.7 V, the current density for lead deposition from PbCl_2_ reaches a maximum of ca. 21.67 and 23.20 mA cm^–2^ on gallium and Galinstan electrodes, respectively, whereas the maximum current density on gallium and Galinstan using Pb(NO_3_)_2_ reaches around 11.77 and 11.29 mA cm^–2^, respectively.

To determine the nucleation and growth mechanism of lead deposition, these chronoamperometry curves can be modeled in terms of plotting  versus . This is based on the electrochemical theoretical nucleation model proposed by Scharifker and Hills (SH model) (eqs 1 and 2), which defines 3D nucleation processes, including both progressive and instantaneous nucleation mechanisms, through the analysis of potentiostatic current transients.

For instantaneous 3D nucleation, the equation is as follows
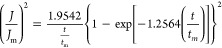
1whereas, the equation for progressive 3D nucleation is
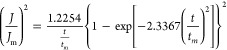
2where *J* is the current density (A cm^–2^) at time *t*, and *J*_m_ is the maximum current density (A cm^–2^) at time *t*_m_.

**Figure 5:**
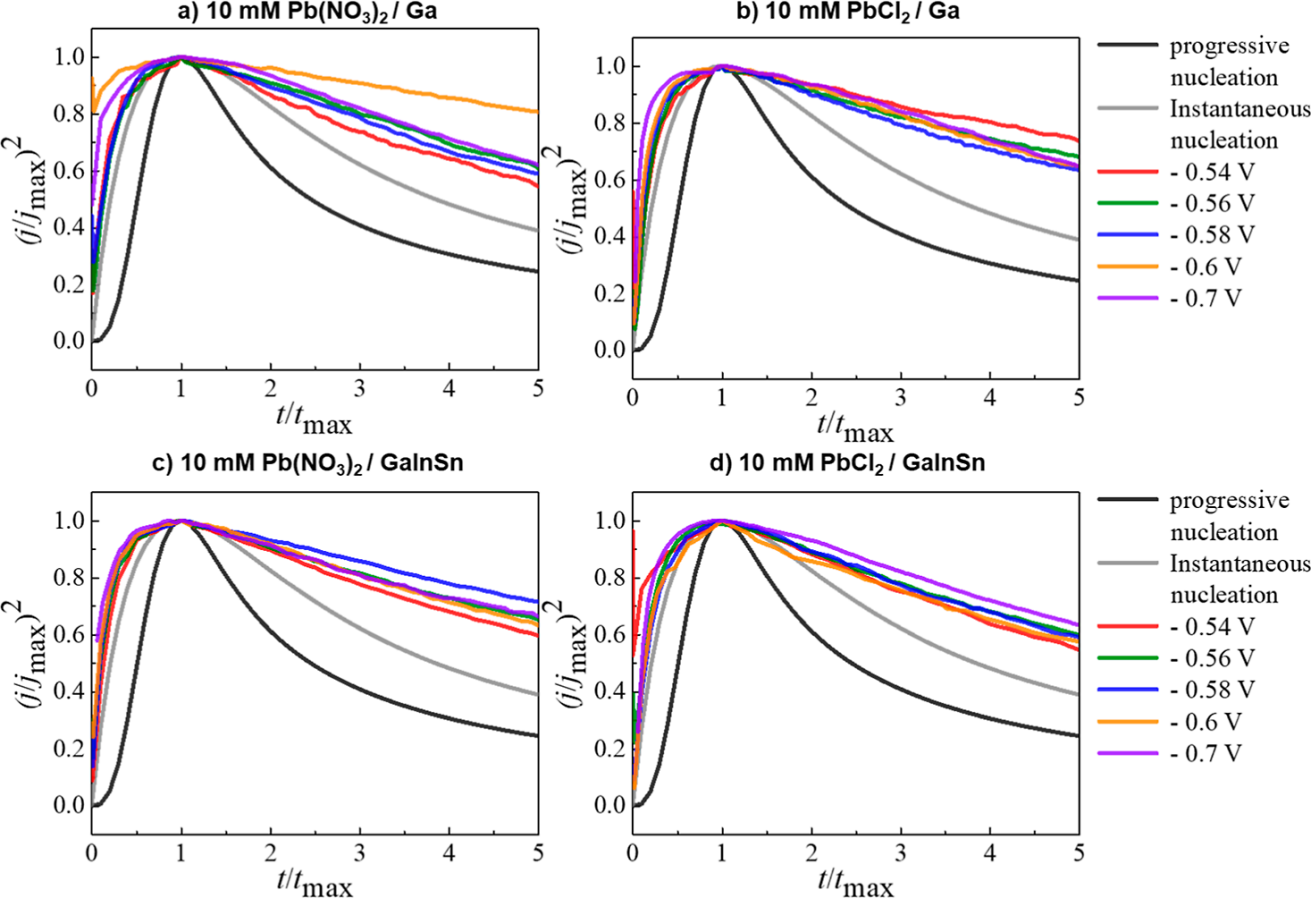
Comparison of the theoretical nondimensional plots of (*j*/*j*_max_)^2^ versus (*t*/*t*_max_) for instantaneous and progressive nucleation against experimental transient current densities at different potentials.

The chronoamperometry results were plotted to compare the relationship between  and  with this theoretical model. For the four electrochemical deposition conditions presented in Figure 5, these plots indicate that the electrodeposition of lead on liquid metal electrodes follows the theoretical model of instantaneous 3D nucleation up to the value of *t*_max_. The risingpart of the experimental transient in Figure 5 is very close to the instantaneous model when deposition potentials are −0.54 and −0.56 V but deviates to higher (*j*/*j*_max_) values after increasing the deposition potential to −0.60 and −0.70 V. This difference is due to the fact that the theory is developed for nuclei with hemispherical geometry,^52^ whereas in this case it is seen from the SEM imaging (Figure 3) that elongated oval shaped particles are formed rather than hemispherical particles. After the transient reaches *t*_max_, the experimental transient exhibited a higher current at longer time after *t*_max_ where such deviation has been attributed to partial kinetic control of the growth,^53^ or mass transport limitations and nuclei overlap.^54^ This type of behavior was observed for the electrodeposition of alkali metals on Hg electrodes where an instantaneous nucleation 2D growth model was proposed whereby a monolayer was not formed but rather that crystals of the new phase grew at different rates in different directions.^54^ This could also be the case here where elongated growth from initially formed seed particles occur (Figure 3).

**Figure 6:**
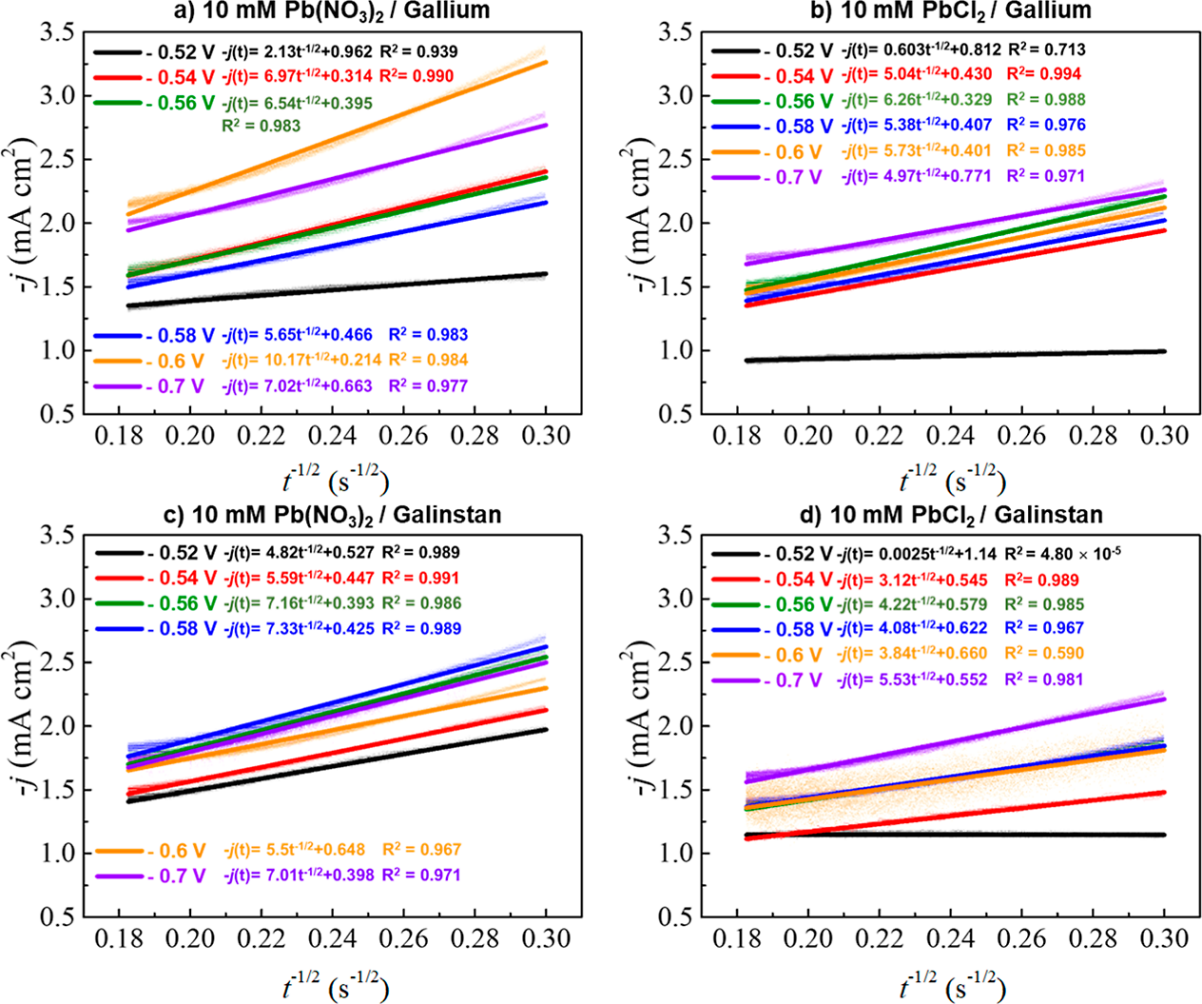
Transients current density (*j*) versus *t*^–1/2^ for the last part of the chronoamperometric curves at different potentials for lead electrodeposition on Ga and GaInSn liquid metal electrodes. This is conducted in a 0.2 M Sodium acetate buffer (pH 4.5) containing 10 mM PbCl_2_ and Pb(NO_3_)_2_.

The diffusion coefficient for Pb^2+^ ions was then calculated from the falling part of the transient using the Cottrell eq 3.
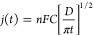
3Here, *n* represents the number of electrons transferred during electrodeposition, *F* is Faraday’s constant (*F* = 96,485 C mol^–1^), *C* is the concentration of the lead salt (mol L^–1^), *D* is the diffusion coefficient (cm^2^ s^–1^), and *t* is time (s). The relationship between the transient current density (*j*) and *t*^–1/2^ is depicted in Figure 6. All transient curves exhibit an expected linear relationship with varying slopes where the slope can be used to calculate *D* for lead deposition in the two electrolytes (Pb(NO_3_)_2_, PbCl_2_) on the two liquid metal electrodes (Gallium and Galinstan), which is summarized in Table 2. The average *D* values for lead deposition on gallium in Pb(NO_3_)_2_ and PbCl_2_ [excluding the potential (*E*) at −0.52 V] are 4.7 × 10^–5^ and 2.5 × 10^–5^ cm^2^ s^–1^, respectively. For lead deposition on Galinstan in Pb(NO_3_)_2_, the average *D* value [excluding the potential (*E*) at −0.52 V] is 3.0 × 10^–5^ cm^2^ s^–1^, and in PbCl_2_, it is 1.5 × 10^–5^ cm^2^ s^–1^. The *D* values calculated from both equations did not increase when the deposition potential was changed to more negative values. This is in agreement with lead deposition on copper electrodes, although the value of *D* is higher compared to the Cu case (0.330 to 0.687 × 10^–5^ cm^2^ s^–1^).^55^ These values indicate that the diffusion coefficient for lead deposition in Pb(NO_3_)_2_ is generally higher than in PbCl_2_ and may be due to the presence of different anions in the electrolyte, while the *D* values calculated when using Ga or GaInSn electrodes are comparable.

**Table 2:** Diffusion Parameters *D* for Lead Electrodeposition on the Liquid Metal Electrodes Ga and GaInSn from a 0.2 M Sodium Acetate Buffer (pH 4.5) Containing Either 10 mM PbCl_2_ or Pb(NO_3_)_2_

*E* (V vs Ag/AgCl)	*D* (10^–5^ cm^2^ s^–1^)
Ga-10 mM PbNO_3_	
–0.52	0.382
–0.54	4.10
–0.56	3.61
–0.58	2.69
–0.6	8.72
–0.7	4.16
GaInSn-10 mM PbNO_3_	
–0.52	1.96
–0.54	2.64
–0.56	4.32
–0.58	4.53
–0.6	2.55
–0.7	4.14
Ga-10 mM PbCl_2_	
–0.52	0.031
–0.54	2.14
–0.56	3.31
–0.58	2.44
–0.6	2.77
–0.7	2.08
GaInSn-10 mM PbCl_2_	
–0.52	5.27 × 10^–7^
–0.54	0.82
–0.56	1.50
–0.58	1.40
–0.6	1.24
–0.7	2.58

Previous work has shown that when Pb and Bi were electrodeposited onto liquid Ga electrodes it resulted in directed growth to form triangles when low concentrations (<2.5 mM) were used for long deposition times of 15 min where an absence of a current maximum in the current vs time transients was noted.^40^ Here at increased concentration of lead salt and reduced electrodeposition times directed growth is still observed which favors the formation of microwires of Pb on both liquid gallium and Galinstan electrodes through an instantaneous nucleation and growth mechanism.

## Conclusion

This research has provided key insights into the electrochemical formation of lead microwires on liquid metal electrodes, focusing on gallium and Galinstan (GaInSn) alloys in sodium acetate buffer containing lead chloride, and lead nitrate electrolytes. These findings revealed through SEM imaging and further characterized by EDS analysis, include the observation of various structures such as wire structure, branch-like and flake-like formations. The research also demonstrated how different media, specifically PbCl_2_ and Pb(NO_3_)_2_, influence the crystallization morphology of lead. Additionally, the study analyzed the effects of electrolyte concentration and deposition time on the morphology of lead electrodeposition, noting important differences in structural development between gallium and Galinstan. The electrochemical analysis within this study provided valuable insights into current density-time transients and diffusion coefficients under various potentials. This analysis enhanced our understanding of the nucleation kinetics and growth mechanisms of lead, closely aligning with the theoretical models of instantaneous nucleation and growth. The research not only deepens our understanding of lead electrodeposition on gallium and Galinstan but also offers potential for future applications in nanoelectronics and sensing devices, where these unique nanostructures could be effectively utilized.

## Supporting Information Available

SEM images of lead electrodeposition and the cyclic voltammogram recorded at varied concentrations of electrolyte (PDF)
