# Supplementary material for: Electrochemical Formation of Pb Microwires with Tunable Morphology on Liquid Metal Electrodes
Source: ACS Omega. 2024 Oct 31;9(45):45641–50. doi: 10.1021/acsomega.4c09165 (PMC11561610; doi:10.1021/acsomega.4c09165)
Supplement: Supplementary file 1 — ao4c09165_si_001.pdf [file ao4c09165_si_001.pdf]

SUPPORTING INFORMATION

Electrochemical Formation of Pb Microwires with Tunable Morphology on Liquid Metal Electrodes

Panjaphong Lertsathitphong[1], Sarunputt Limpijumnong[1], Mithran Somasundrum[2], Anthony P. O'Mullane[3], Benchaporn Lertanantawong[1]*

[1] Biosensors Laboratory, Department of Biomedical Engineering, Faculty of Engineering, Mahidol University, Nakhon Pathom, 73170, Thailand

[2] Biosciences and System Biology Team, Biochemical Engineering and System Biology Research Group, National Center for Genetic Engineering and Biotechnology, National Science and Technology Development Agency at KMUTT, Bangkok, 10150 Thailand

[3] School of Chemistry, Physics and Mechanical Engineering, Queensland University of Technology (QUT), Brisbane, QLD 4001, Australia

Table of Contents

SEM and EDS element mapping images in $NaNO_3$ p.2

Cyclic voltammogram at varying concentrations of lead electrolyte p.3

SEM images at varying concentrations of lead electrolyte. p.3

Additional SEM images at varying lead deposition time p.4

Optical images of lead electrodeposition using GaInSn electrode p.4

Corresponding Author

*Email: benchaporn.ler@mahidol.ac.th

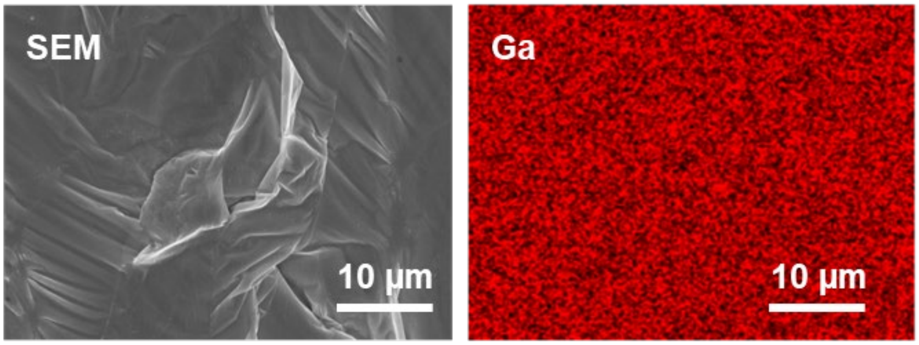

Figure S1 SEM and EDS element mapping images of lead electrodeposition in a 0.2 M sodium acetate buffer (pH 4.5) containing 10 mM $NaNO_3$, using Gallium electrode.

The effect of lead salt concentration and deposition time was investigated to provide insights into the initial stages of lead deposition and subsequent microwire formation. As illustrated in Supplementary Figure S2, the electrochemical behavior varies with the concentration of the lead salt that is used (in this data for $PbCl_2$ is shown). As expected with an increasing concentration of lead salt the deposition and oxidation peaks shift to more negative and positive potentials, respectively accompanied by an increase in current passed for each process which is highly consistent between both liquid metal electrodes and is typical behaviour that is also seen for metal deposition on solid electrodes. The presence of a crossover or nucleation loop at all concentrations indicates nucleation and growth kinetics are involved.

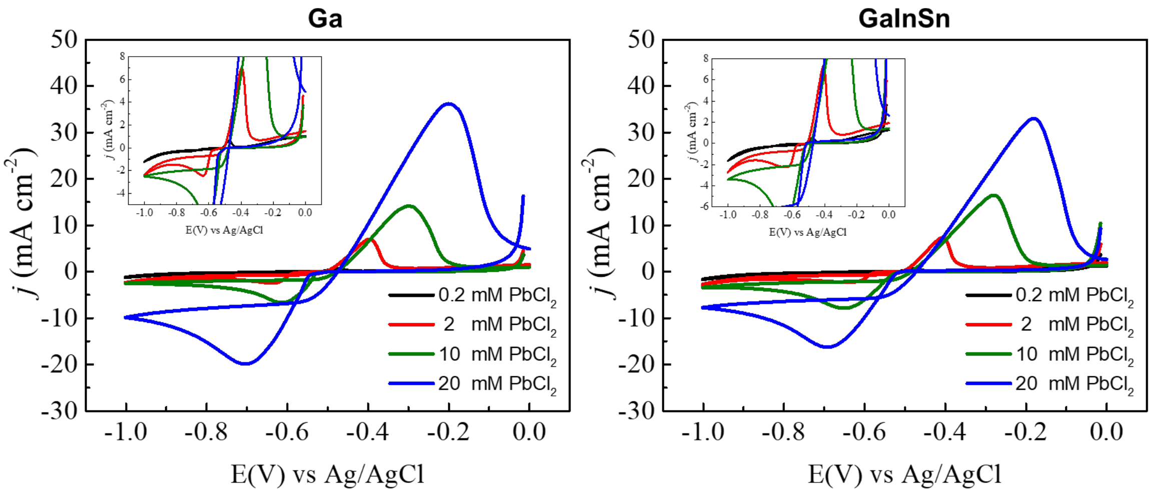

Figure S2. The first cyclic voltammogram recorded at liquid gallium and galinstan electrodes in a 0.2 M Sodium acetate buffer (pH 4.5), containing $PbCl_2$ at varying concentrations.

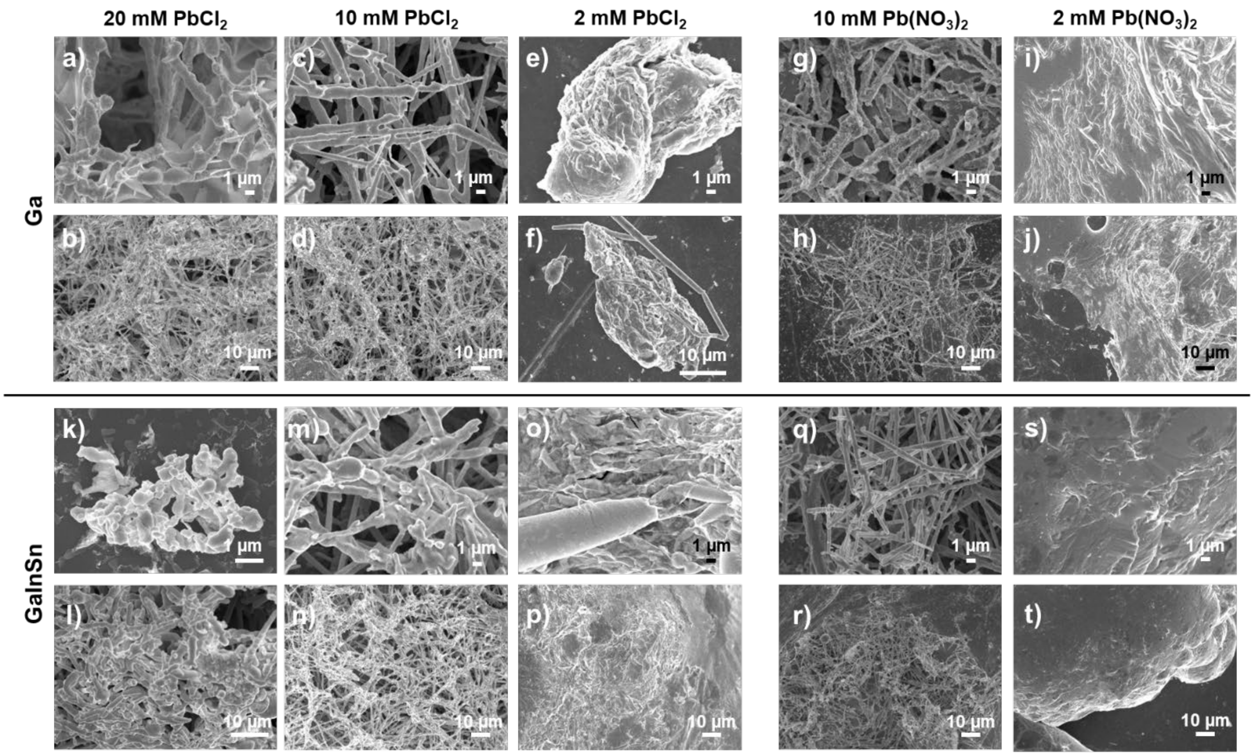

Figure S3. SEM images of lead electrodeposition on Gallium (a-j) and Galinstan (k-t) liquid metal electrodes in a 0.2 M sodium acetate buffer (pH 4.5) containing varying concentrations of $PbCl_2$: 20 mM (a, b, k, l), 10 mM (c, d, m, n), 2 mM (e, f, o, p), and and $Pb(NO_3)_2$: 10 mM (g, h, q, r), 2 mM (i, j, s, t).

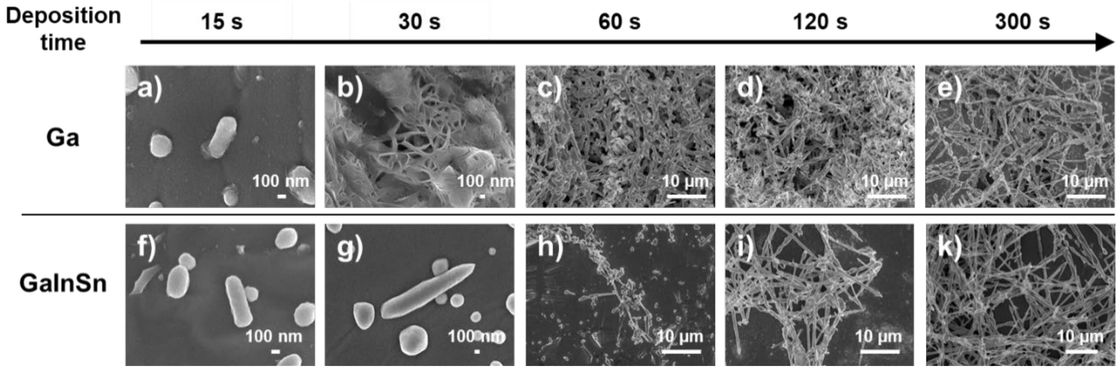

Figure S4. Additional SEM images of lead electrodeposition from a 0.2 M Sodium acetate buffer (pH 4.5) containing 10 mM $Pb(NO_3)_2$ on Ga (a-e) and GaInSn (f-k) liquid metal electrodes over varying deposition times: (a, f) 15 sec, (b, g) 30 sec, (c, h) 60 sec, (d, i) 120 sec, and (e, k) 300 sec.

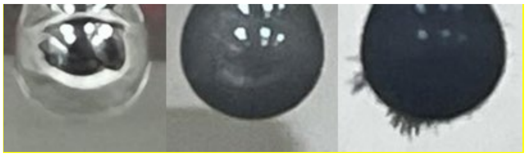

Figure S5. Optical images of lead electrodeposition using GaInSn electrode in a 0.2 M sodium acetate buffer (pH 4.5) containing 10 mM $Pb(NO_3)_2$, over varying deposition times at 0, 60 and 300 second respectively, from left to right.
